# Supplementary material for: Identification of photoperiod-induced specific miRNAs in the adrenal glands of Sunite sheep (Ovis aries)
Source: Front Vet Sci. 2022 Jul 22;9:888207. doi: 10.3389/fvets.2022.888207 (PMC9354845; doi:10.3389/fvets.2022.888207)
Supplement: Supplementary file 1 [file Data_Sheet_1.ZIP › Supplementary file/miRDeep2 file.zip!/miRDeep2 file/Know_Structure/SP42A2/oar-mir-376d.pdf]

miRBase precursor	: oar-mir-376d
Total read count	: 203
oar-miR-376d read count	: 198
remaining reads	: 5

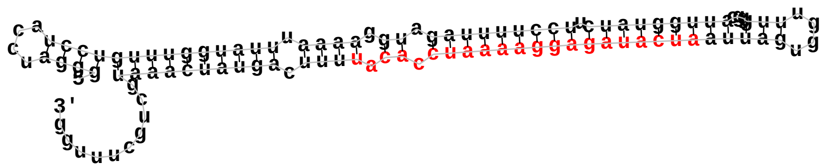

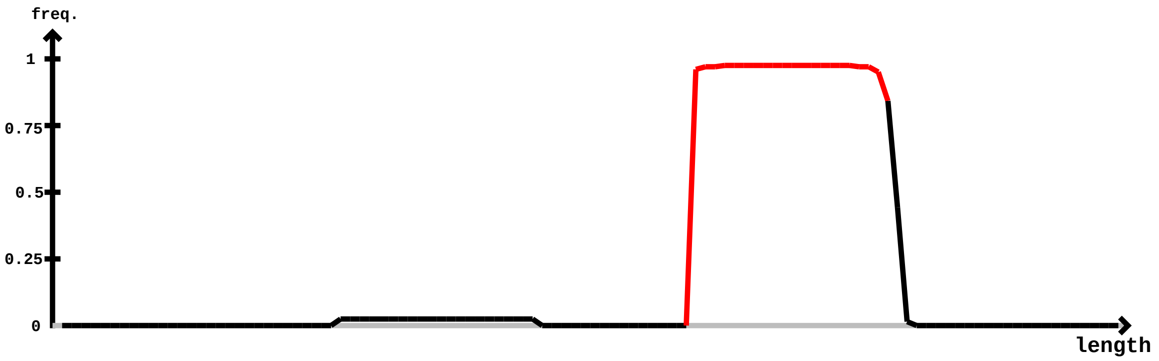

oar-miR-376d

```
5'- gggauccauccuguuugguauuuaaaagguagauuuuccuucuaugguuacggguuuggugauuaaucauagaggaaaauccacauuucaguaucaaaugcugcuuugg   -3'    exp
    ((((....))))((((((((((.((((.((.(((((((((.(((((((((...(((....))))))))))))))))))))).)).)))).))))))))))..........   reads      mm         sample
    ............................guagauuuuccuucuaugguu............................................................   5          0          seq
    .................................................................aucauagaggaaaaucc...........................   1          0          seq
    .................................................................aucauagaggaaaauccac.........................   4          0          seq
    .................................................................aucauagaggaaaauccaca........................   22         0          seq
    .................................................................aucauagaggaaaauccacau.......................   81         0          seq
    .................................................................aucauagaggaaaauccacauu......................   85         0          seq
    .................................................................aucauagaggaaaauccacauuu.....................   2          0          seq
    ..................................................................ucauagaggaaaauccacauu......................   1          0          seq
    ..................................................................ucauagaggaaaauccacauuu.....................   1          0          seq
    ....................................................................auagaggaaaauccacauu......................   1          0          seq
```
